# The domestication of SARS-CoV-2 into a seasonal infection by viral variants

**DOI:** 10.3389/fmicb.2023.1289387

**Published:** 2023-12-20

**Authors:** Ryley D. McClelland, Yi-Chan James Lin, Tyce N. Culp, Ryan Noyce, David Evans, Tom C. Hobman, Vanessa Meier-Stephenson, David J. Marchant

**Affiliations:** ^1^Department of Medical Microbiology and Immunology, University of Alberta, Edmonton, AB, Canada; ^2^Department of Cell Biology, University of Alberta, Edmonton, AB, Canada; ^3^Department of Medicine, University of Alberta, Edmonton, AB, Canada

**Keywords:** seasonal infection, virus, adaptation, COVID19, coronavirus, SARS, surrogates

## Abstract

**Introduction:**

The COVID-19 pandemic was caused by the zoonotic betacoronavirus SARS-CoV-2. SARS-CoV-2 variants have emerged due to adaptation in humans, shifting SARS-CoV-2 towards an endemic seasonal virus. We have termed this process ‘virus domestication’.

**Methods:**

We analyzed aggregate COVID-19 data from a publicly funded healthcare system in Canada from March 7, 2020 to November 21, 2022. We graphed surrogate calculations of COVID-19 disease severity and SARS-CoV-2 variant plaque sizes in tissue culture.

**Results and Discussion:**

Mutations in SARS-CoV-2 adapt the virus to better infect humans and evade the host immune response, resulting in the emergence of variants with altered pathogenicity. We observed a decrease in COVID-19 disease severity surrogates after the arrival of the Delta variant, coinciding with significantly smaller plaque sizes. Overall, we suggest that SARS-CoV-2 has become more infectious and less virulent through viral domestication. Our findings highlight the importance of SARS-CoV-2 vaccination and help inform public policy on the highest probability outcomes during viral pandemics.

## Introduction

Domestication refers to the act of gradual adaptation and taming towards humans such that the organism is genetically distinct from its wild counterparts. There are many accepted definitions of domestication and many whose wording applies to the description of our evolving relationship with the SARS-CoV-2 virus. Like many domesticated animal species, the term “domestication” does not equate to safety; both domesticated animals and seasonal viruses like influenza and respiratory syncytial virus are significant burdens to global mortality each year (Forrester et al., 2018; Paget et al., 2019; Du et al., 2023).

The spillover of the SARS-CoV-2 virus from bats through an unknown intermediate species into the human population precipitated the COVID-19 pandemic (Worobey et al., 2022). Prior to COVID-19, coronavirus outbreaks of SARS-CoV and MERS-CoV occurred in 2003 and 2012, respectively. The 2003 SARS-CoV is no longer circulating in humans and human MERS-CoV cases occur sporadically. The propensity of SARS-CoV-2 to rapidly spread within the human population is linked to its ability to be transmitted by infected persons before symptoms develop and by those who are asymptomatic.

We expect the eventual domestication of SARS-CoV-2 to occur akin to seasonal human coronaviruses (HCoV) such as HKU1, OC43 and NL63. These seasonal coronaviruses share distinct similarities in genome sequence, receptor usage and biology of infection. Indeed, HCoV-OC43 likely originated as a zoonotic bovine coronavirus that became seasonal in humans due to a pandemic that occurred in 1890 (Vijgen et al., 2005). We believe that immune processes such as vaccination and immune memory will continue to drive the emergence of SARS-CoV-2 variants. This serves as an indication that domestication of SARS-CoV-2 is underway and will eventually become another endemic seasonal infection like HCoV-OC43.

In this study, we provide support that SARS-CoV-2 emerged into humans as a more virulent infection that has reduced in pathogenesis with an increase in infectiousness over time, coinciding with the emergence and dominance of SARS-CoV-2 variants of concern (VOCs). We warn that this process could make long-COVID more difficult to diagnose as infection becomes insidious and there is an increased risk of misdiagnosis. We have called this process of “virus domestication.”

## Methods

### Data sources

This was a population-based longitudinal study using publicly available data on COVID-19 case statistics and vaccination rates in Alberta retrieved from the Government of Alberta website. The Canadian Province of Alberta has a publicly funded and publicly accessible health care system. Throughout the pandemic the Government of Alberta has recorded case, hospitalization, ICU admission and deaths due to SARS-CoV-2 infection and made the data publicly available. In this data set, there was 618,030 cumulative COVID-19 cases and 5,177 cumulative deaths attributed to COVID-19 in Alberta. The number of new hospitalizations and ICU admissions were recorded daily.

### Calculation of surrogate markers of COVID-19 disease severity

The daily ICU admissions and deaths from aggregate data on COVID-19 cases in Alberta were divided by the hospitalizations at each respective day and tabulated.

### Culture and plaque assay of SARS-CoV-2 variants

Culture of SARS-CoV-2 variants has been described previously with the following modifications: Vero E6/TMPRSS2 was used instead of Vero CCL-81 cells (Lin et al., 2022). Results are the product of one experiment but are representative of at least three independent replicate experiments per strain. Plaque sizes were measured using ImageJ (NIH) and reported as plaque area (mm^2^).

### Statistical analysis

Plaque diameters were measured and reported as plaque area (mm^2^). Statistical significance between plaque areas was by one-way ANOVA with Tukey’s test of significance using Graphpad Prism 10.

## Results

**Figure 1:**
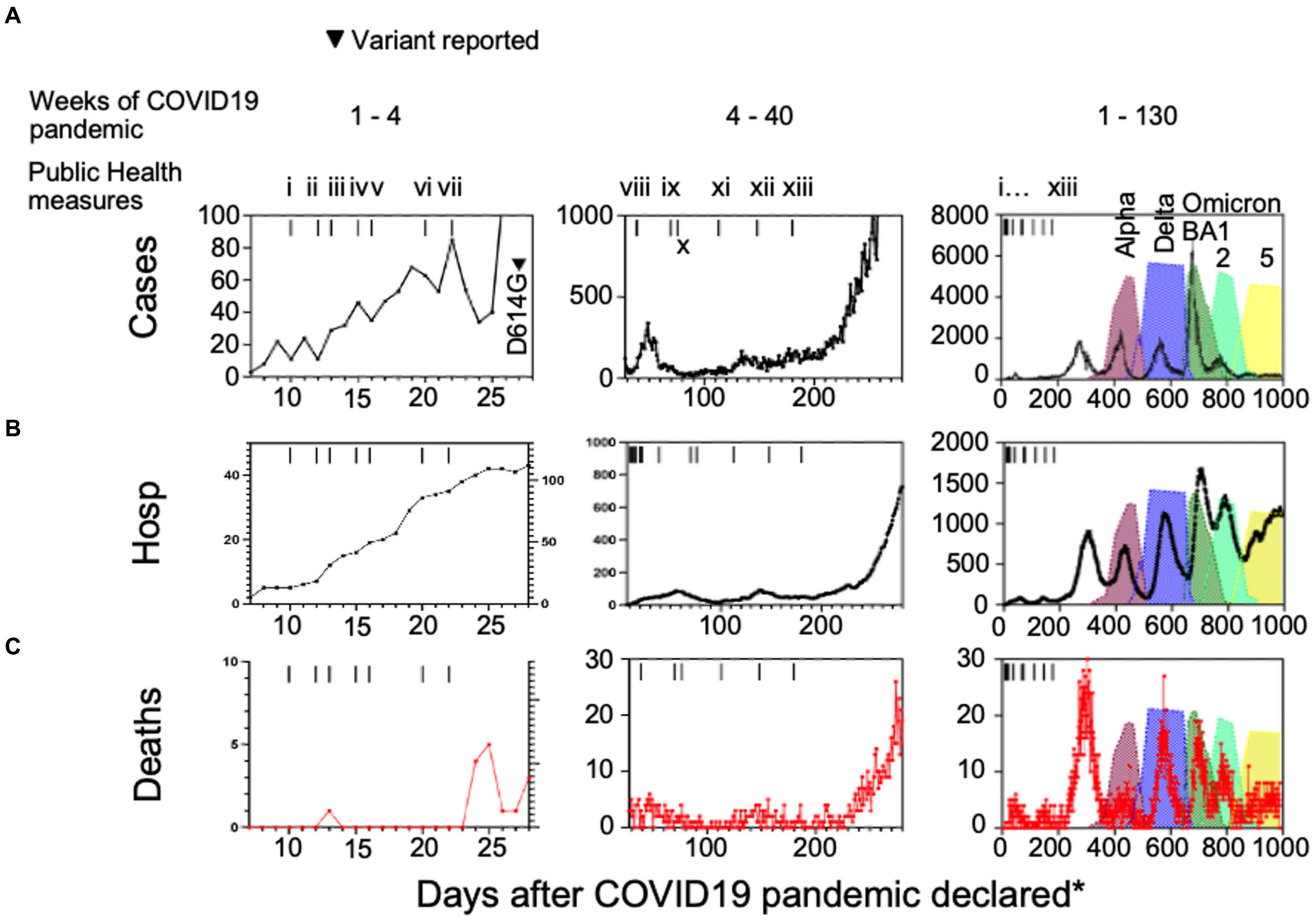
**(A)** Case numbers, **(B)** hospitalizations, and **(C)** deaths compared to public health measures and variants of interest introduced in Alberta, Canada. (i) school closures province-wide, (ii) public health emergency declared in Alberta, (iii) non-emergency health care procedure cancellations, (iv) care home lockdown, (v) US-Canada border closed, (vi) quarantine measures for international travelers introduced, (vii) restaurant and retail closures, (viii) test all who are symptomatic, (ix) mask wearing advised in Alberta and (x) Canada-wide, (xi) 2 m social distancing in public places, (xii) masks mandatory in public settings across Canada, (xiii) in-class grades kindergarten to 12 resume with masking, hand hygiene, and social distancing protocols. Hosp = hospitalizations, ^*^days after the COVID-19 pandemic was declared by the World Health Organization on March 7th, 2020.

We charted cases, hospitalizations, ICU admissions and deaths due to COVID-19 in the Canadian province of Alberta (Figure 1). Aggregate data show the detection of SARS-CoV-2 cases in the general population after March 7th, 2020 when Alberta Clinical Laboratories began testing for SARS-CoV-2 by PCR (Pabbaraju et al., 2022; Stokes et al., 2022). The World Health Organization declared a pandemic on March 11th, 2020. Figure 1 shows that there was an initial rise in daily infections, hospitalizations, and deaths, that then decreased with the enactment of COVID-19 health protection laws (Figure 1; i to x). In the weeks following the reopening of schools for in-class instruction after week 28 (day 200; health measure xiii) of the pandemic there was a wave of cases, hospitalizations, and deaths that occurred.

### The arrival of variants coincided with a decrease in surrogate indicators of COVID-19 severity

**Figure 2:**
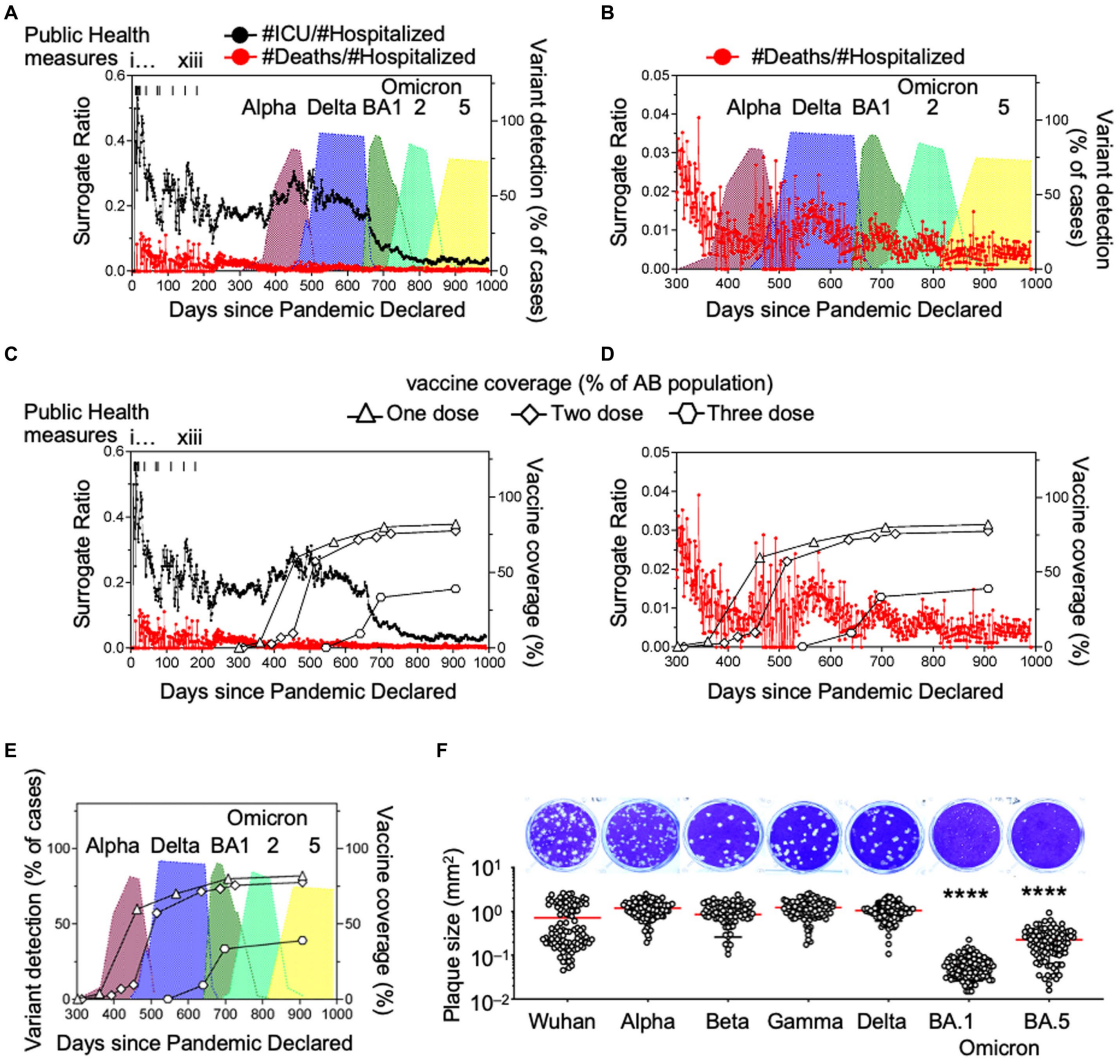
Ratios of daily hospitalizations to ICU admissions and daily hospitalizations to deaths as surrogate markers of COVID-19 disease severity and *in vitro* plaque sizes of variants. **(A,B)** Daily ICU admissions or deaths due to SARS-CoV-2 were divided by daily hospitalizations due to SARS-CoV-2 to illustrate the association of the death surrogate with variants Alpha, Gamma, Delta and Omicron introduction in Alberta, Canada and **(C–E)** association with vaccination rate with one, two, and three doses of SARS-CoV-2 vaccine. **(F)** SARS-CoV-2 isolates of each variant were plated on cells simultaneously with methylcellulose overlay, stained and imaged 3 days later. Diameters of the plaques were measured and reported as plaque area (mm²). Significance was determined by one-way ANOVA with Tukey’s test of significance. ^*^p < 0.0001.

Consistent with the domestication model, we observed a decrease in daily deaths (Figure 1C) whereas daily cases increased to their highest levels with the peak of Omicron BA1 prevalence (Figure 1A). We postulated that this was an indicator of mean reduced pathogenesis in the population, so we defined surrogates of COVID-19 disease severity. Here we chose hospitalization as the denominator because ICU cases and deaths would often be recorded as hospitalization initially, prior to ICU admission or death. We divided the daily number of intensive care unit (ICU) admissions or deaths (Figures 2A–D) by the number of hospitalized cases, so the calculation serves as an approximation of COVID-19 severity within a one-week to one-month time frame of hospital admission. There was a considerable reduction in ICU admissions versus hospitalizations coinciding with the arrival of variants Delta and Omicron, in particular.

### Surrogate pathogenicity coincided with VOC plaque sizes

Viral escape from immune pressure and increased infectivity are the result of mutations in the VOCs, but these mutations come at a cost to viral replication. In a cell monolayer, the size of the virus plaques are related to the replicative fitness of the virus (Mandary et al., 2020). Consistently, we previously reported that respiratory syncytial virus (RSV) plaque sizes were associated with RSV viral load in patient samples (Elawar et al., 2017). Since SARS-CoV-2 viral loads are related to disease severity (Fajnzylber et al., 2020), we asked if SARS-CoV-2 plaque size was associated with disease surrogates of COVID-19. In a cell monolayer of Vero E6/TMPRSS2 cells, VOC plaque sizes were measured using ImageJ and reported as plaque area (mm^2^) (Figure 2F). As we move from wild type to Delta, plaque size increased, relating to increased viral production and therefore improved replicative fitness. This coincided with the increased infectivity seen at the population level (Hui et al., 2022). This was paired with evidence of stronger spike protein affinity leading to higher viral loads, potentially explaining its greater infectivity in the population (Kissler et al., 2021; Mannar et al., 2022). The spike proteins of Delta and Omicron variants have similar binding affinities for ACE2 (Mannar et al., 2022). However, we found that Omicron plaque sizes were significantly smaller than Delta plaque sizes (Figure 2F). This suggests that a process distinct from attachment is impacting viral fitness and leading to less viral replication in VOCs (Mannar et al., 2022). As the COVID-19 pandemic progressed and mutations arose from immune escape, we observed a coincident change in behavior in cell culture, including a reduction in variant plaque sizes as variants emerged over time. We noted that the ICU: hospitalization surrogates and plaque sizes decreased by about 10-fold and 17-fold with Omicron as compared to Delta variants, respectively (Figures 2A,B,F).

### Vaccination efficacy, variants, and COVID-19 severity surrogates

COVID-19 vaccinations have prevented considerable morbidity and mortality worldwide. Having antibodies at the ready, whether through vaccination or natural infection has clear advantages at the individual level to clear the infection more readily. Average viral loads between vaccinated and unvaccinated individuals have not been shown to be significantly different in acute infections (Kissler et al., 2021). Studies on Omicron have suggested that it can evade most pre-existing neutralizing antibodies though some indicate that broadly neutralizing antibodies maintain their efficacy (Cameroni et al., 2022). Though the mutations of SARS-CoV-2 that affect receptor affinity and antibody sensitivity have been accurately and exhaustively studied, many of these mutations are compensatory so it is very difficult to ascribe these traits to any one mutation (Moulana et al., 2022).

We suggest that our COVID-19 disease severity surrogates can serve as indicators of population immunity. To determine whether the emergence of the variants affected disease severity we graphed Alberta vaccination rates (for the purpose of completeness) with ICU and death surrogate ratios (Figures 2C,D). From these data, we noted that the emergence of Delta and other SARS-CoV-2 variants in Alberta coincided with one and two dose vaccination rates of 60% and 57.3%, respectively (Figure 2E).

The Delta variant arrived after en-masse vaccination on day 501 and the first Omicron BA1 strain arrived on day 648 with BA2 and BA5 arriving on days 713 and 835, respectively. Vaccination coverage with 1 and 2 doses were 82% and 77.6% of the population, respectively, by August 29th 2022 (day 907). If we were to incorporate immunity from the natural infection itself, these numbers would be even higher.

Consistent with our observation, others observed a 91% decrease in mortality with Omicron infections compared to the Delta variant (Lewnard et al., 2022). Though our data does not consider immunity at the individual level, if there was immune escape by a mutation or set of mutations, we expect that ICU admissions and death surrogates would have increased after arrival of Delta and Omicron (Andrews et al., 2022).

## Discussion

The emergence of seasonal coronaviruses from their pandemic predecessors suggests that a virus domestication process occurs. That is, mutations adapt the virus to infect humans and evade the host immune response at the cost of replicative fitness and pathogenicity. This adaptation process is evident in the emergence of SARS-CoV-2 VOCs. Mutations in SARS-CoV-2 have resulted in increased human ACE2 receptor affinity at the cost of the rate of replication (Moulana et al., 2022) and higher tropism for the upper respiratory tract (Hui et al., 2022) that may represent the domestication process toward a less severe but more common seasonal infection. Importantly, the reduced pathogenicity of SARS-CoV-2 could make long-term COVID-19 sequelae more insidious, harder to diagnose and therefore highlights the increased importance of vaccination.

As we report here, mutation and outgrowth of variants is a normal part of virus domestication and overall leads to less pathogenic outcomes in the population over time. At this point we do not know if SARS-CoV-2 has become completely domesticated into a seasonal virus. However, we predict that this will likely be the point at which hospitalized cases occur primarily during Winter months, coinciding with other seasonal respiratory viruses. We outline these principles to help inform on highest probability outcomes during viral pandemics. In the face of virus mutation, these principles may help inform public policy.

## Data availability statement

The data presented in this study were retrieved from the Government of Alberta website. The dataset contains no potential identifiers and is available in the Supplementary files.

## Ethics statement

The studies involving humans were approved by University of Alberta Human Research Ethics Board 2. The studies were conducted in accordance with the local legislation and institutional requirements. Written informed consent for participation was not required from the participants or the participants’ legal guardians/next of kin in accordance with the national legislation and institutional requirements.

## Author contributions

RM: Conceptualization, Data curation, Formal analysis, Investigation, Methodology, Visualization, Writing – original draft, Writing – review & editing. Y-CL: Data curation, Methodology, Writing – review & editing. TC: Conceptualization, Investigation, Methodology, Writing – review & editing. RN: Conceptualization, Writing – review & editing. DE: Writing – review & editing. TH: Conceptualization, Writing – review & editing. VM-S: Conceptualization, Visualization, Writing – original draft, Writing – review & editing. DM: Conceptualization, Data curation, Funding acquisition, Investigation, Methodology, Supervision, Writing – original draft, Writing – review & editing.

## References

[ref1] Andrews N. Stowe J. Kirsebom F. Toffa S. Rickeard T. Gallagher E.. (2022). Covid-19 vaccine effectiveness against the Omicron (B.1.1.529) variant. N. Engl. J. Med. 386, 1532–1546. doi: 10.1056/NEJMoa2119451, PMID: 35249272 PMC8908811

[ref2] Cameroni E. Bowen J. E. Rosen L. E. Saliba C. Zepeda S. K. Culap K.. (2022). Broadly neutralizing antibodies overcome SARS-CoV-2 Omicron antigenic shift. Nature 602, 664–670. doi: 10.1038/s41586-021-04386-2, PMID: 35016195 PMC9531318

[ref3] Du Y. Yan R. Wu X. Zhang X. Chen C. Jiang D.. (2023). Global burden and trends of respiratory syncytial virus infection across different age groups from 1990 to 2019: a systematic analysis of the global burden of disease 2019 study. Int. J. Infect. Dis. 135, 70–76. doi: 10.1016/j.ijid.2023.08.008, PMID: 37567553

[ref4] Elawar F. Griffiths C. D. Zhu D. Bilawchuk L. M. Jensen L. D. Forss L.. (2017). A virological and phylogenetic analysis of the emergence of new clades of respiratory syncytial virus. Sci. Rep. 7:12232. doi: 10.1038/s41598-017-12001-6, PMID: 28947776 PMC5612963

[ref5] Fajnzylber J. Regan J. Coxen K. Corry H. Wong C. Rosenthal A.. (2020). Massachusetts consortium for pathogen, SARS-CoV-2 viral load is associated with increased disease severity and mortality. Nat. Commun. 11:5493. doi: 10.1038/s41467-020-19057-5, PMID: 33127906 PMC7603483

[ref6] Forrester J. A. Weiser T. G. Forrester J. D. (2018). An update on fatalities due to venomous and nonvenomous animals in the United States (2008–2015). Wilderness Environ. Med. 29, 36–44. doi: 10.1016/j.wem.2017.10.004, PMID: 29373216

[ref7] Hui K. P. Y. Ho J. C. W. Cheung M. C. Ng K. C. Ching R. H. H. Lai K. L.. (2022). SARS-CoV-2 Omicron variant replication in human bronchus and lung *ex vivo*. Nature 603, 715–720. doi: 10.1038/s41586-022-04479-6, PMID: 35104836

[ref8] Kissler S. M. Fauver J. R. Mack C. Tai C. G. Breban M. I. Watkins A. E.. (2021). Viral dynamics of SARS-CoV-2 variants in vaccinated and unvaccinated persons. N. Engl. J. Med. 385, 2489–2491. doi: 10.1056/NEJMc2102507, PMID: 34941024 PMC8693673

[ref9] Lewnard J. A. Hong V. X. Patel M. M. Kahn R. Lipsitch M. Tartof S. Y. (2022). Clinical outcomes associated with SARS-CoV-2 Omicron (B.1.1.529) variant and BA.1/BA.1.1 or BA.2 subvariant infection in Southern California. Nat. Med. 28, 1933–1943. doi: 10.1038/s41591-022-01887-z, PMID: 35675841 PMC10208005

[ref10] Lin Y. C. Malott R. J. Ward L. Kiplagat L. Pabbaraju K. Gill K.. (2022). Detection and quantification of infectious severe acute respiratory coronavirus-2 in diverse clinical and environmental samples. Sci. Rep. 12:5418. doi: 10.1038/s41598-022-09218-5, PMID: 35354854 PMC8967087

[ref11] Mandary M. B. Masomian M. Ong S. K. Poh C. L. (2020). Characterization of plaque variants and the involvement of quasi-species in a population of EV-A71. Viruses 12:651. doi: 10.3390/v12060651 32560288 PMC7354493

[ref12] Mannar D. Saville J. W. Zhu X. Srivastava S. S. Berezuk A. M. Tuttle K. S.. (2022). SARS-CoV-2 Omicron variant: antibody evasion and cryo-EM structure of spike protein-ACE2 complex. Science 375, 760–764. doi: 10.1126/science.abn7760, PMID: 35050643 PMC9799367

[ref13] Moulana A. Dupic T. Phillips A. M. Chang J. Nieves S. Roffler A. A.. (2022). Compensatory epistasis maintains ACE2 affinity in SARS-CoV-2 Omicron BA.1. Nat. Commun. 13:7011. doi: 10.1038/s41467-022-34506-z, PMID: 36384919 PMC9668218

[ref14] Pabbaraju K. Zelyas N. Wong A. Croxen M. A. Lynch T. Buss E.. (2022). Evolving strategy for an evolving virus: development of real-time PCR assays for detecting all SARS-CoV-2 variants of concern. J. Virol. Methods 307:114553. doi: 10.1016/j.jviromet.2022.114553, PMID: 35644262 PMC9134755

[ref15] Paget J. Spreeuwenberg P. Charu V. Taylor R. J. Iuliano A. D. Bresee J.. (2019). Global mortality associated with seasonal influenza epidemics: new burden estimates and predictors from the GLaMOR project. J. Glob. Health 9:020421. doi: 10.7189/jogh.09.020421, PMID: 31673337 PMC6815659

[ref16] Stokes W. Berenger B. M. Scott B. Szelewicki J. Singh T. Portnoy D.. (2022). One swab fits all: performance of a rapid, antigen-based SARS-CoV-2 test using a nasal swab, nasopharyngeal swab for nasal collection, and RT-PCR confirmation from residual extraction buffer. J. Appl. Lab. Med. 7, 834–841. doi: 10.1093/jalm/jfac004, PMID: 35258088 PMC8992337

[ref17] Vijgen L. Keyaerts E. Moes E. Thoelen I. Wollants E. Lemey P.. (2005). Complete genomic sequence of human coronavirus OC43: molecular clock analysis suggests a relatively recent zoonotic coronavirus transmission event. J. Virol. 79, 1595–1604. doi: 10.1128/JVI.79.3.1595-1604.2005, PMID: 15650185 PMC544107

[ref18] Worobey M. Levy J. I. Malpica Serrano L. Crits-Christoph A. Pekar J. E. Goldstein S. A.. (2022). The Huanan seafood wholesale market in Wuhan was the early epicenter of the COVID-19 pandemic. Science 377, 951–959. doi: 10.1126/science.abp8715, PMID: 35881010 PMC9348750

